# Healthcare Mobility between East and West, Two Forthcoming Challenges

**Published:** 2017-10

**Authors:** Ladan ROKNI, Ji-Yeong YUN

**Affiliations:** 1. Dept. of Tourism Management, Eastern Mediterranean University, Famagusta, North Cyprus; 2. Body Culture Institut, Konkuk University, Seoul, South Korea

## Dear Editor-in-Chief

Medical tourism has been emerged due to the trend of healthcare mobility among different countries. In fact it is a new type of healthcare mobility (1, 2) since not only it involves the clinical services, but also other non-clinical services are likely to be essential and also it is highly affected by cultural factors (3). Eastern countries or developing countries are mostly the main destination for western countries or more affluent societies due to several reasons discussed in previous studies.

Such a trend from west to east caused variety of issues to be discussed and study by the authorities and researchers. The characters of healthcare system differ based on the regulation of each country, from the other perspective, patients are in their “most physically and emotionally vulnerable” situation (4) and it provides added pressure to tolerate cultural differences in this situation (5). Provided that medical tourists are neither patient nor tourist (asserted by the website of MTQA) the process of providing an appropriate service in medical tourism is likely to be much more striking.

Considering the cultural background of demand and supply sides, two main challenges can be discussed. The first challenge implies on the cultural differences of doctors and patients, while the second challenge comes from the fact that many immigrants travel back to their homeland for medical services, mainly because of cultural similarities. Both these trends lead to a wide range of questions and demand for clarification.

### Cultural Competence

Although medical tourism has been emerged due to the globalization in healthcare and the main treatment procedures are similar all around the world, cultural differences between the eastern and western continents, even among each country, potentially can lead to some barriers of interaction between doctors and patients (6). Lack of cultural competency among healthcare practitioners might lead to misdiagnosis (7) and potentially influence on their relationship. Accordingly it is essential to deliver a service to medical tourist which is congruent to their culturally and personally needs.

Cultural competence in facts implies on the “knowledge” about cultural and personal background of patients which is transformed into “skills” and service approaches as well. It is a learned system (8) but lack of a uniform procedure can be seen to deliver cultural competence within the healthcare context; hence there is a demand of focusing on an appropriate and effective process and delivery of cultural competence from doctors to foreign patients, specifically in the scope of medical tourism which is a novel domain with a wide range of ambiguities. Professional training and supports is required to train specialized manpower with the ability of delivering a “culturally oriented patient-centered care” (4). Accordingly, medical tourism is likely to be a key domain which is highly affected by these differences, while a dearth of information in this arena demands for more practical research.

### Immigrants and Home Medical Service

Cultural background and cultural similarities has been considered among the key motivation factors for patients-tourists in the process of choosing a destination for their treatment. Traveling back home for immigrants provides a sense of socially and culturally belonging; the other reason is system familiarity since the healthcare system differs based on the regulation of each country. Returning home with medical propose not only is the matter of treatment, but also it implies on the level of trust that patients has to their national doctors, hospitals and all the treatment procedures. It is believed that those who travel back home for medical treatments are mainly in the search of more wellbeing which can be provided through an “in place trust and familiarity” (9). It gives the immigrants a sense of control over their health.

Accordingly, migrants prefer to return their country of origin with the aim of treatment in order to have an appropriate health system to their culture and their social construction as well.

Although so far the issue of immigrants and equal access to culturally diverse healthcare has been considered by the researchers and authorities from different disciplines, nevertheless traveling back home for treatment is likely to cause further ambiguities for the researchers. Several considerable facts in this regard are as follows:

1. It is not clear that those patients should be considered as medical tourists or national patients,
2. The type of insurance that they use might lead to several problems for the authorities,
3. It is not clear how they should be treated about tax payment, similar to national people or international tourists,
4. Although the patients and doctors have almost similar cultural background, there are still some specific requirements and demands that differ from the national patients,
5. The issue of doctor’s safety is not clear since this group patients are neither local people nor international.

It is likely that a different strategy is required in medical tourism according to the differences between “patient-consumers” as a tourist and those “immigrants-patients-costumers” who prefer to travel back to their country of origin for treatment; the former groups just cross the borders oftentimes for having medical care, however the latter follow the similar process while they have a similar cultural background with the doctors and are living outside the country. Accordingly, it is essential to clarify the regulations regarding those immigrants who travel back to their country of origin for treatment.
